# Carbon monoxide poisoning increases T_peak_–T_end_ dispersion and QT_c_ dispersion

**DOI:** 10.5830/CVJA-2014-012

**Published:** 2014-06

**Authors:** Murat Eroglu, Ali Osman Yildirim, Omer Uz, Zafer Isilak, Murat Yalcin, Ejder Kardesoglu

**Affiliations:** Department of Emergency Medicine, Haydarpasa Teaching Hospital, Gulhane Military Medical Academy, Istanbul, Turkey; Department of Emergency Medicine, Haydarpasa Teaching Hospital, Gulhane Military Medical Academy, Istanbul, Turkey; Department of Cardiology, Haydarpasa Teaching Hospital, Gulhane Military Medical Academy, Istanbul, Turkey; Department of Cardiology, Haydarpasa Teaching Hospital, Gulhane Military Medical Academy, Istanbul, Turkey; Department of Cardiology, Haydarpasa Teaching Hospital, Gulhane Military Medical Academy, Istanbul, Turkey; Department of Cardiology, Haydarpasa Teaching Hospital, Gulhane Military Medical Academy, Istanbul, Turkey

**Keywords:** carbon monoxide, electrocardiogram, dysrhythmia, ventricular repolarisation

## Abstract

**Objective:**

Carbon monoxide (CO) poisoning leads to cardiac dysrhythmia. Increased heterogeneity in ventricular repolarisation on electrocardiogram (ECG) shows an increased risk of arrhythmia. A number of parameters are used to evaluate ventricular repolarisation heterogeneity on ECG. The aim of our study is to investigate the effect of acute CO poisoning on indirect parameters of ventricular repolarisation on ECG.

**Methods:**

Sixty-seven patients were included in this case–control study. Thirty patients with acute CO poisoning were assigned to group 1 (19 females, mean age: 30.8 ± 11.3 years). A control group was formed with patients without known cardiac disease (group 2, *n* = 37; 25 females, mean age: 26.0 ± 5.2 years). Twelve-lead ECG and serum electrolyte levels were recorded in all patients. Also, carboxyhaemoglobin (COHb) levels were recorded in group 1. T_peak_–T_end_ (T_p_T_e_) interval, T_p_T_e_ dispersion, T_p_T_e_/QT ratio, QT interval and QT_d_ durations were measured as parameters of ventricular repolarisation. Corrected QT (QT_c_) and QT_c_ dispersion (QT_cd_) intervals were determined with the Bazett’s formula.

**Results:**

The mean COHb level in group 1 was 27.6 ± 7.4% and mean duration of CO exposure was 163.5 ± 110.9 min. No statistically significant difference was found in age, gender, serum electrolytes or blood pressure levels between the groups. QRS, QT, QT_c_, T_p_T_e_ interval and T_p_T_e_/QT ratio were similar between the groups (*p* > 0.05). QTcd (65.7 ± 64.4 vs 42.1 ± 14.2 ms, *p* = 0.003) and T_p_T_e_ dispersion (40.5 ± 14.8 vs 33.2 ± 4.9 ms, *p* = 0.006) were significantly longer in group 1 than group 2. COHb level was moderately correlated with TpTe dispersion (*r* = 0.29; *p* = 0.01).

**Conclusion:**

To our knowledge, this is the first study to investigate T_p_T_e_ interval and dispersion in CO poisoning. Our results showed that T_p_T_e_ dispersion and QTc dispersion increased after CO poisoning.

## Abstract

Carbon monoxide (CO) poisoning may cause myocardial toxicity and life-threating cardiac arrhythmias.^1^-^3^ Acute coronary syndrome, myocardial injury, myocardial dysfunction, cardiac arrest and various types of arrhythmias have been reported in patients with acute CO poisoning.^4^ CO binds myocardial myoglobin and reduces myocardial oxygen reserve.^5^ Previous studies reported that episodes of atrial fibrillation, premature ventricular beats and sinusal tachycardia may be seen in patients with acute CO poisoning.^6^,^7^ Recent studies also suggested that risk of atrial and ventricular arrhythmia is increased in CO poisoning, due to prolonged QT_c_ and QT_c_ dispersion.^2^,^3^,^8^

Ventricular repolarisation can be evaluated by measuring QT interval, corrected QT interval, and QT dispersion. Among these parameters, QT dispersion represents the heterogeneity of ventricular repolarisation and was clearly shown to be associated with ventricular arrhythmia.^9^ T_peak_–T_end_ (T_p_T_e_) interval is defined as the interval between the peak point and endpoint of the T wave on surface electrocardiography and is a novel index of transmural dispersion of ventricular repolarisation.^10^ T_p_T_e_/QT ratio and T_p_T_e_/QT_c_ ratio were used in previous studies as an electrocardiographic index in the evaluation of risk of ventricular arrhythmia.^11^,^12^

The effect of acute CO poisoning on QT intervals was investigated in a number of studies.^2^,^3^,^8^ However, to the best of our knowledge, T_p_T_e_ interval, T_p_T_e_ dispersion, T_p_T_e_/QT ratio and T_p_T_e_/QT_c_ ratio have not been investigated sufficiently in patients with CO poisoning. In this study, we aimed to investigate the effect of acute CO poisoning on electrocardiographic parameters, which indirectly show ventricular repolarisation heterogeneity. We also investigated the relationship between carboxyhaemoglobin (COHb) levels and these parameters.

## Methods

The ethics committee of Gulhane Military Medical Academy Haydarpasa Teaching Hospital approved the study protocol. The control group was composed of 37 healthy medical staff or volunteers aged from 20 to 40 years (mean 26.0; SD = 5.2), comprising 25 women and 12 men. Patients who were treated with normobaric oxygen for CO poisoning at the Emergency Department of Gulhane Military Medical Academy between 1 October 2005 and 31 May 2006 comprised the study group. Diagnosis of CO poisoning was made based on medical history and a COHb level > 5% (10% in smokers).

Patients excluded from the study were those with coronary artery disease or other known heart disease, such as valvular diseases or rhythm disorders, those taking drugs known to influence QT interval, patients with ECG abnormalities such as atrial fibrillation, conduction delay, bundle branch blocks, immeasurable T waves, and those with stroke, obstructive lung diseases, malignancies and those who received hyperbaric oxygen therapy.

On admission to the emergency department, blood samples were obtained for blood gas analysis, total blood cell counts and biochemical parameters. COHb measurements were performed with Synthesis 45 (Italy).

Baseline 12-lead ECGs were recorded with a paper speed of 25 mm/s and standardisation of 1.0 mV/cm in all patients. The QT intervals were measured from the onset of the QRS complex to the end of the T wave, defined as the return T-P baseline. When U waves were present, the QT intervals were measured to the nadir of the notch between the T and U waves. QT_c_ interval was calculated using the Bazett’s formula. The QT_c_ dispersion (QT_cd_) is the difference between minimum and maximum QT_c_ intervals.

T_p_T_e_ interval was measured from the peak of the T wave to the end of the T wave. The end of the T wave was defined as the junction of the T wave with the isoelectric line. The difference between minimum and maximum T_p_T_e_ intervals on ECG (T_p_T_e.max_–T_p_T_e.min_) was considered T_p_T_e_ dispersion. T_p_T_e_/QT ratio and T_p_T_e_/QT_c_ ratio were also calculated. Two experienced cardiologists (ZI and MY), who were unaware of the patient’s clinical condition, took two measurements of the QT and T_p_T_e_ interval from each measurable lead.

## Statistical analysis

The data are presented as mean ± SD. The independent-samples *t*-test was used to compare continuous variables and the chi-square test was used for categorical variables. Pearson’s correlation coefficients were determined for the relationship of COHb levels with ECG parameters (QT_c_, QT_cd_, T_p_T_e_, T_p_T_e_ dispersion and T_p_T_e_/QT_c_). A *p*-value < 0.05 was accepted as statistically significant. Statistical analyses were performed using SPSS 11.0 (SPSS Inc., Chicago, IL).

## Results

A total of 67 patients (28.5 ± 9.0 years, 44 female) were included in the study. Eight (27%) among the CO-intoxicated patients were smokers. Clinical characteristics of the patients are presented in Table 1. Mean COHb level was 27.6 ± 7.4%. Mean duration of CO exposure was 164 ± 111 minutes and mean emergency department arrival time was 68 ± 123 minutes. We found a negative correlation between the time to emergency department arrival and COHb level (*r* = –0.568, *p* = 0.001). We also found a negative correlation between age and COHb level (*r* = –0.469, *p* = 0.01).

**Table 1:** Clinical characteristics of the study population.

	*CO-intoxicated patients (n = 30)*	*Normal subjects (n = 37)*	p*
Age (years)	30.8 ± 11.3	26.0 ± 5.2	> 0.05
Gender (F/M)	19/11	25/12	> 0.05
BMI (kg/m^2^)	23.1 ± 5.5	24.6 ± 6.9	> 0.05
Mean heart rate (beats/min)	92.5 ± 16.2	82.0 ± 13.0	> 0.05
SBP (mmHg)	118.7 ± 9.6	122.1 ± 8.7	> 0.05
DBP(mmHg)	78.2 ± 8.4	72.1 ± 7.5	> 0.05
CO exposure time (min)	163.5 ± 110.9		
COHb (g/dl)	27.6 ± 7.4		
Time to ED arrival (min)	68.3 ± 123.1		
Smoker, *n* (%)	8 (27)	11 (30)	> 0.05

BMI, body mass index; SBP, systolic blood pressure; DBP, diastolic blood pressure; ED, emergency department.

Seven patients among the CO-intoxicated patients had sinus tachycardia on the ECG records taken at the emergency department. The mean heart rate of the CO-intoxicated patients was found to be mildly higher than that of the normal subjects. However, the difference was not statistically significant (*p* > 0.05) (Table 1).

The QT_cd_ durations of CO-intoxicated patients were significantly longer than that of normal subjects (63.1 ± 10.9 vs 42.1 ± 4.3 ms; *p* = 0.0001) (Table 2). The QTcd value was detected to be above 60 ms in 19 subjects of the CO-intoxicated patients (63%) and in none of the normal subjects (*p* < 0.001).

**Table 2:** Electrocardiographic measurements of the groups.

	*CO-intoxicated patients (n = 30)*	*Normal subjects (n = 37)*	p*
QT interval (ms)	355.7 ± 90.7	359.6 ± 26.4	0.51
QT_c_ interval (ms)	382.1 ± 11.4	403.7 ± 19.7	0.31
T_p_T_e_ /QT_c_ time (ms)	0.26 ± 0.02	0.20 ± 0.02	0.16
T_p_T_e_/QT_d_ time (ms)	1.78 ± 0.32	1.85 ± 0.27	0.2
T_p_T_e_/QT_cd_ time (ms)	1.52 ± 0.29	2.0 ± 0.34	0.001
T_p_T_e_ dispersion (ms)	41.4 ± 13.0	33.2 ± 4.9	0.001
T_p_T_e_/QT time (ms)	0.26 ± 0.04	0.23 ± 0.02	0.11
QT_d_ interval (ms)	57.2 ± 10.8	55.1 ± 3.7	0.1
QT_cd_ interval (ms)	63.1 ± 10.9	42.1 ± 4.3	0.0001
T_p_T_e_ time (ms)	87.5 ± 19.0	83.1 ± 8.3	0.21

The T_p_T_e_ dispersion value of the CO-intoxicated patients was significantly higher than that of normal subjects (41.4 ± 13.0 vs 33.2 ± 4.9 ms; *p* = 0.001). TpTe/QTcd ratio was lower in the CO-intoxicated patients compared to the normal subjects (1.52 ± 0.29 vs 2.0 ± 0.34; *p* = 0.001).

**Fig. 1.**
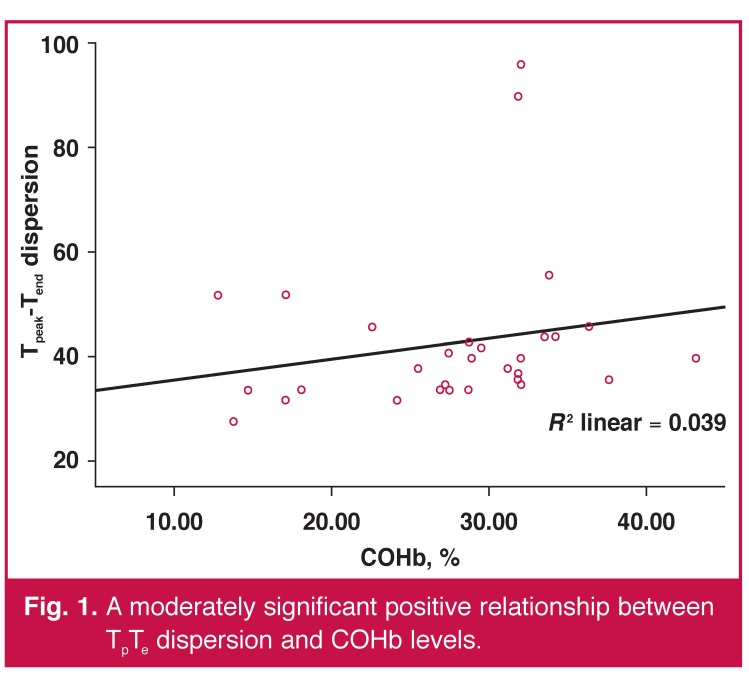
A moderately significant positive relationship between T_p_T_e_ dispersion and COHb levels.

Pearson’s correlation analysis revealed that a moderately significant positive correlation was present only between T_p_T_e_ dispersion and COHb levels (*r* = 0.39, *p* = 0.03) (Fig. 1). Correlations between electrocardiographic measurements and COHb levels of the patients are presented in Table 3.

**Table 3:** Correlations between electrocardiographic measurements and COHb levels.

	*R*	p*
QT interval (ms)	–0.12	0.52
QT_c_ interval (ms)	–0.11	0.53
QT_d_ interval (ms)	0.07	0.68
QT_cd_ interval (ms)	0.18	0.33
T_p_T_e_ time (ms)	0.19	0.33
T_p_T_e_ dispersion (ms)	0.39	0.03*
T_p_T_e_/QT time (ms)	0.08	0.66
T_p_T_e_ /QT_c_ (ms)	0.17	0.35
T_p_T_e_ /QT_d_ (ms)	0.06	0.71
T_p_T_e_ /QT_cd_ (ms)	0.07	0.69

## Discussion

Our results showed that T_peak_–T_end_ dispersion and QT_c_ dispersion were higher in CO-intoxicated patients compared to normal subjects. T_p_T_e_/QTcd ratio was lower in CO-intoxicated patients compared to normal subjects. We found a positive correlation only between T_peak_–T_end_ dispersion and COHb level. Our results indicated that T_p_T_e_ dispersion may be one of the reasons for arrhythmia caused by CO poisoning.

CO may lead to persistent or reversible myocardial damage, mainly due to myocardial hypoxaemia and direct action of CO on the heart.^13^ Binding to myoglobin may reduce oxygen availability in the heart and cause arrhythmias and cardiac dysfunction.^14^ Cardiovascular effects of CO poisoning include tachycardia, hypotension, dysrhythmia, ischaemia, infarction, and, in some cases, cardiac arrest.^15^,^16^ Previous studies reported that episodes of atrial fibrillation, premature ventricular beats and sinus tachycardia developed in patients with acute CO poisoning.^6^,^7^

QT and QT_c_ show ventricular repolarisation on ECG. A prolonged QT interval indicates impaired myocardial refractoriness. Prolonged QT and QT_c_ intervals can cause a number of arrhythmias, including torsades de pointes, polymorphic ventricular tachycardia and ventricular fibrillation.^17^,^18^ A number of studies have investigated the effect of acute CO poisoning on QT and QT_c_ intervals. These studies found that QT_c_ but not QT interval was prolonged in CO-poisoned patients compared to control subjects.^4^,^19^ In our study, however, we found that neither QT nor QT_c_ intervals was prolonged after CO poisoning.

QT and QT_c_ dispersion represent physiological variability of regional ventricular repolarisation. Increased QT and QT_c_ dispersions are related to heterogeneity of regional ventricular repolarisation and are accepted as the markers of arrhythmias.^17^,^20^ Data concerning the effect of acute CO poisoning on QT and QT_c_ dispersion is limited. However, it has been reported that CO poisoning increased QT and QTc dispersion.^4^,^19^ We found that the durations of QT_cd_ were significantly prolonged in adult patients with acute CO poisoning.

T_p_T_e_ interval is used as an index of transmural dispersion of ventricular repolarisation.^10^ T_p_T_e_ dispersion, T_p_T_e_/QT ratio and T_p_T_e_/QTc ratio are also used as an electrocardiographic index of ventricular arrhythmogenesis.^12^,^21^ Sicouri *et al.* found a relationship between ventricular arrhythmia and prolonged T_p_T_e_ interval.^22^

Previous studies have demonstrated that prolongation of T_p_T_e_ duration is associated with increased mortality in Brugada syndrome, long QT syndromes, hypertrophic cardiomyopathy, and in patients undergoing primary percutaneous coronary intervention for myocardial infarction.^11^ In our study, T_p_T_e_ interval, T_p_T_e_/QT ratio and T_p_T_e_/QTc ratio did not change significantly after CO poisoning. However, we did find a correlation between T_p_T_e_ dispersion and COHb levels.

In our study we found that only QTc dispersion and T_p_T_e_ dispersion increased in patients with CO poisoning. We concluded that these two parameters are more valuable among the ECG parameters to demonstrate risk of ventricular arrhythmia in patients with CO poisoning.

The limitation of this study was the relatively small number of patients with CO poisoning. Therefore, a follow-up investigation with a larger sample size is warranted.

## Conclusion

Our results showed that T_peak_–T_end_ dispersion and QT_c_ dispersion increased after CO poisoning. We believe that CO poisoning impaired the homogeneity of ventricular repolarisation and may have caused increased T_peak_–T_end_ dispersion and QT_c_ dispersion. Further studies are needed to evaluate the importance of electrocardiographic parameters in CO poisoning.
